# Visual performance and patient-reported outcomes of a non-apodized diffractive trifocal intraocular lens in Chinese cataract patients: a prospective multicenter real-world study

**DOI:** 10.3389/fmed.2026.1853791

**Published:** 2026-07-08

**Authors:** Yan Wen, Qiong Liao, Shuai Chen, Maosheng Chen, Xiaocheng Zhang, Lei Wu, Zhengdong Xu, Li An, Jilin Tan

**Affiliations:** 1 Department of Cataract Surgery, Chongqing Aier Eye Hospital, Chongqing, China; 2 Chongqing Eye and Vision Care Hospital, Chongqing, China; 3 West China Hospital, Sichuan University, Chengdu, Sichuan, China; 4 Chongqing Aier Mega Eye Hospital, Chongqing, China; 5 Chengdu Aier Eye Hospital, Chengdu, Sichuan, China; 6 Chengdu East Aier Eye Hospital, Chengdu, Sichuan, China; 7 Kunming IWE Eye Hospital, Kunming, Yunnan, China

**Keywords:** cataract, patient satisfaction, presbyopia, real-world study, trifocal intraocular lens, visual outcomes

## Abstract

**Purpose:**

To assess clinical visual performance and patient-reported satisfaction following bilateral implantation of a non-apodized diffractive trifocal intraocular lens (IOL), the AcrySof^®^ IQ PanOptix^®^ Trifocal IOL, in a real-world clinical setting in China.

**Methods:**

This was a prospective, multicenter registry study including patients aged ≥ 18 years with bilateral age-related or complicated cataracts who underwent bilateral implantation of the PanOptix IOL. The primary endpoint was the proportion of patients achieving binocular uncorrected distance (UDVA), intermediate (UIVA), and near (UNVA) visual acuity of ≤0.200 logMAR at 3 months postoperatively. Secondary endpoints included best-corrected distance visual acuity (BCDVA), binocular defocus curves ranging from +2.00 D to −4.00 D, refractive predictability, and patient-reported outcomes assessed using the Catquest-9SF and IOLSAT questionnaires. The study was registered at Chinese Clinical Trial Registry (ChiCTR2100052791; 2021-11-05).

**Results:**

A total of 137 patients completed the 3-months postoperative follow-up. At 3 months, the mean binocular UDVA, UIVA, and UNVA were 0.017 ± 0.086, 0.061 ± 0.110, and 0.061 ± 0.103 logMAR, respectively. Overall, 91.24% of patients achieved the composite endpoint of ≤0.200 logMAR binocularly at all three distances. The binocular defocus curve demonstrated a broad plateau of visual acuity, maintaining ≤0.200 logMAR between +0.50 D and −2.50 D. Refractive predictability was high, with 84.62% of eyes within ±0.50 D of emmetropia. Complete spectacle independence was reported by 96.90% of patients.

**Conclusion:**

Bilateral implantation of the PanOptix trifocal IOL provides excellent and early stable visual performance at distance, intermediate, and near in Chinese cataract patients. High refractive predictability and a high rate of spectacle independence are associated with substantial patient satisfaction in real-world clinical practice.

## 1 Introduction

Cataract remains the leading cause of reversible visual impairment and blindness worldwide, with its prevalence increasing markedly with advancing age (1). The conventional management of cataract has predominantly consisted of phacoemulsification followed by implantation of a monofocal intraocular lens (IOL), which reliably restores distance visual acuity but does not correct presbyopia (2). As a result, patients frequently continue to rely on spectacles for intermediate and near tasks, which can substantially affect postoperative quality of life in a modern environment that is increasingly dependent on digital devices (3). The reconceptualization of cataract surgery as a refractive procedure has driven the development of a range of presbyopia-correcting IOLs designed to provide an extended or continuous range of vision (4).

Diffractive trifocal IOLs constitute a major advancement in this domain by generating three principal foci for far, intermediate, and near vision (5). In contrast to earlier bifocal designs, which frequently resulted in suboptimal intermediate vision, trifocal lenses are intended to improve functional performance at intermediate distances relevant to computer use and interaction with handheld electronic devices (6). The PanOptix model is a non-apodized diffractive trifocal IOL that incorporates proprietary ENLIGHTEN optical technology (7). This technology redistributes light energy from the first diffractive intermediate order to the distance focus while preserving an intermediate focal distance of 60 cm and a near focal distance of 40 cm. The optical design is intended to optimize light utilization and provide a more continuous and natural range of vision for contemporary daily activities (8, 9).

Although the clinical efficacy of the PanOptix IOL has been demonstrated in multiple controlled trials, there remains a need for large-scale, prospective, real-world evidence, particularly in Chinese populations (10, 11). Real-world studies yield critical information on lens performance across heterogeneous clinical settings and patient populations, which may differ substantially from the tightly regulated conditions of randomized controlled trials. Variables such as surgeon-dependent technique, preoperative ocular status, and postoperative neuroadaptation can exhibit considerable variability in routine clinical practice (12, 13). Moreover, the specific visual demands and lifestyle characteristics of Chinese patients, including preferred working distances and ergonomic habits for reading and computer use, necessitate focused assessment of visual performance at distances such as 60 and 40 cm (14, 15).

Accordingly, this prospective multicenter registry study was designed to systematically evaluate visual outcomes, refractive predictability, and patient-reported satisfaction following bilateral implantation of the PanOptix IOL in a prospective multicenter cohort of Chinese patients undergoing cataract surgery in routine clinical practice. By characterizing outcomes in a real-world context, this study aims to provide evidence to support clinical decision-making, patient selection, and preoperative counseling for trifocal IOL implantation.

## 2 Materials and methods

This investigation was designed as a prospective, multicenter registry study conducted in routine ophthalmic practice at tertiary eye institutions in Southwest China, including centers located in Chongqing, Chengdu, and Kunming. Although the study was observational and registry-based, the eligibility criteria reflected standard clinical selection for bilateral trifocal IOL implantation. Patients with ocular comorbidities that could independently compromise postoperative visual acuity, contrast sensitivity, or patient-reported satisfaction were excluded to avoid attributing comorbidity-related visual impairment to the implanted IOL. Eyes with regular corneal astigmatism ≥ 1.00 D were not included because a toric trifocal IOL model was not available in the participating centers during the study period. The study protocol received approval from the Institutional Review Board of Chongqing Aier Eye Hospital (Approval No: IRB 2021004) and was implemented in accordance with the ethical principles outlined in the Declaration of Helsinki. All participants received comprehensive information regarding the study objectives, procedures, potential risks, and benefits, and provided written informed consent prior to undergoing any study-related examinations or interventions. The study was registered at Chinese Clinical Trial Registry (ChiCTR2100052791; 2021-11-05).

The study population comprised patients aged 18 years or older with a diagnosis of bilateral age-related or complicated cataracts who were scheduled to undergo bilateral implantation of the PanOptix trifocal IOL. Eligible participants were required to have preoperative regular corneal astigmatism of less than 1.00 diopter (D) and to be scheduled for second-eye surgery within 1 month following first-eye surgery. Patients were excluded if they presented with preexisting ocular comorbidities that could compromise postoperative visual outcomes, including but not limited to corneal scarring, amblyopia, glaucoma, age-related macular degeneration, or other retinal pathologies. Additional exclusion criteria comprised a history of corneal refractive surgery and participation in concurrent clinical trials that could confound the evaluation of visual or refractive outcomes.

Surgical procedures were performed by experienced cataract surgeons using either standard microincision phacoemulsification or femtosecond laser-assisted cataract surgery (FLACS). A clear corneal incision was employed. IOL power was calculated using optical biometry and the Barrett Universal II formula, with emmetropia targeted in both eyes. The AcrySof^®^ IQ PanOptix^®^ trifocal IOL (Model TFNT00, Alcon Vision LLC, Fort Worth, TX, USA) was implanted in the capsular bag after cortical clean-up. Postoperative management consisted of a standard regimen of topical antibiotics and anti-inflammatory agents administered for several weeks.

Follow-up examinations were scheduled at 1 day, 1 week, 1 month, and 3 months postoperatively. At each visit, uncorrected distance visual acuity (UDVA) at 5 m, uncorrected intermediate visual acuity (UIVA) at 60 cm, and uncorrected near visual acuity (UNVA) at 40 cm were assessed using logarithm of the minimum angle of resolution (logMAR) charts. Best-corrected distance visual acuity (BCDVA) at 5 m was also recorded, as well as distance-corrected intermediate visual acuity (DCIVA) at 60 cm and distance-corrected near visual acuity (DCNVA) at 40 cm. Subjective refraction and intraocular pressure (IOP) were evaluated at all time points. At the 3-months visit, a binocular defocus curve was obtained by providing the patient with best-corrected distance refraction and sequentially introducing spherical lenses in 0.50-diopter (D) steps from +2.00 D to −4.00 D.

Patient-reported outcomes were assessed using validated questionnaires, including the Catquest-9SF to evaluate vision-related functioning and the IOLSAT to assess spectacle independence and overall satisfaction. The Catquest-9SF questionnaire quantified difficulties in performing daily activities and overall satisfaction with visual function, whereas the IOLSAT questionnaire documented the frequency of spectacle use at different distances and the patient’s willingness to recommend the implanted IOL.

The primary composite endpoint was defined as the proportion of patients achieving binocular uncorrected visual acuity of 0.200 logMAR or better at all three distances (distance, intermediate, and near) at the 3-months follow-up visit.

Sample size calculation. The planned registry sample size was determined based on the desired precision for estimating the primary composite endpoint, defined as the proportion of patients achieving binocular UDVA, UIVA, and UNVA of 0.200 logMAR or better at 3 months. Assuming an expected endpoint achievement rate of 90%, a two-sided 95% confidence level, and an absolute precision of 5%, at least 139 evaluable patients were required according to the single-proportion formula *n* = Z^2^p(1 − p)/d^2^. After allowing for an approximately 20% rate of incomplete follow-up or incomplete case-report documentation, the target enrollment was approximately 173 patients. The enrollment of 175 patients was therefore considered adequate for the planned precision-based analysis.

Statistical analyses were conducted using SPSS software (version 26.0, IBM Corp, Armonk, NY, USA). Continuous variables were summarized as mean ± standard deviation. The normality of data distribution was assessed using the Kolmogorov-Smirnov test. Longitudinal comparisons of visual outcomes were performed using repeated-measures analysis of variance (ANOVA), with Greenhouse-Geisser correction applied when the sphericity assumption was violated. A *p*-value < 0.05 was considered indicative of statistical significance.

## 3 Results

A total of 175 patients were enrolled in the registry, of whom 137 completed the scheduled 3-months postoperative follow-up and were included in the primary analysis. The completion rate was 78.29%, and the incomplete follow-up rate was 21.71%. The main practical reasons for incomplete follow-up were incomplete case-report documentation at some participating centers and failure to attend the scheduled 3-months visit, partly because some patients were less willing to return after satisfactory early postoperative recovery. The study cohort consisted of 58 males (42.34%) and 79 females (57.66%). The mean age was 62.37 ± 11.23 years, with a median age of 61.00 years (interquartile range, 53.00–69.00 years; range, 36–91 years). Overall, 73 patients (53.28%) were aged < 65 years and 64 patients (46.72%) were aged ≥ 65 years (Table 1).

**TABLE 1:** Baseline demographic and preoperative ocular characteristics before PanOptix IOL implantation.

Variable	Value
Patients included in primary analysis, *n*	137
Age, years, mean ± SD	62.37 ± 11.23
Age, years, median (IQR)	61.00 (53.00–69.00)
Age range, years	36–91
Age < 65 years, *n* (%)	73 (53.28%)
Age ≥ 65 years, *n* (%)	64 (46.72%)
Male, *n* (%)	58 (42.34%)
Female, *n* (%)	79 (57.66%)

To evaluate potential attrition bias, we compared baseline characteristics between patients who completed the 3-months follow-up and non-completers with available baseline data. Baseline records were available for 32 of the 38 non-completers; source records for 6 patients could not be fully retrieved because of personnel changes and incomplete case-report documentation at one participating center. Among patients with available baseline data, no statistically significant differences were observed between completers and non-completers in the compared demographic and clinical variables. Because baseline data were unavailable for a small subset of non-completers, the possibility of residual attrition bias cannot be fully excluded.

**FIGURE 1:**
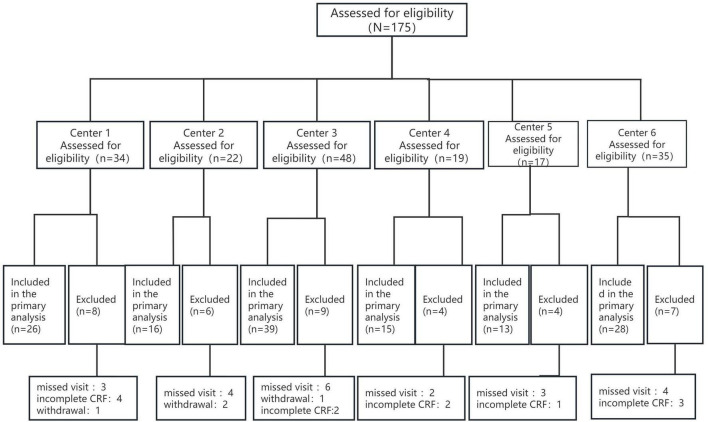
Participant flow diagram stratified by study center. Because this was an observational registry study conducted in routine clinical practice, no random allocation was performed. The diagram summarizes the numbers of patients assessed for eligibility, excluded with reasons, included in the primary analysis, and lost to or not completing the 3-months follow-up with reasons at each participating center. CRF, Case Report Form.

A center-stratified participant flow diagram is provided in Figure 1. Overall, 175 patients were assessed for eligibility across the six participating centers, and 137 patients completed the scheduled 3-months follow-up and were included in the primary analysis. The numbers included in the primary analysis from Centers 1 to 6 were 26, 16, 39, 15, 13, and 28, respectively. The main reasons for incomplete follow-up were missed 3-months visits and incomplete case-report documentation at participating centers.

Preoperative visual and biometric assessments showed mean UDVA values of 0.800 ± 0.480 logMAR in right eyes and 0.890 ± 0.490 logMAR in left eyes. The mean axial length was 24.45 ± 1.77 mm in right eyes and 24.49 ± 1.82 mm in left eyes (Table 2).

**TABLE 2:** Preoperative ocular parameters and visual analysis before PanOptix IOL implantation.

Parameter	Eyes (*n*)	OD		OS	
		Mean	SD	Mean	SD
UDVA 5 m	83	0.80	0.48	0.89	0.49
UIVA 60 cm		0.67	0.41	0.71	0.43
UNVA 40 cm		0.68	0.41	0.72	0.40
Axial length (mm)	101	24.45	1.77	24.49	1.82
Anterior chamber depth (mm)		3.26	0.44	3.29	0.45
Mean keratometry (D) (k1+k2)/2		44.08	1.53	44.07	1.61
Total corneal spherical aberration (μm)	97	0.28	0.16	0.29	0.17
Total corneal higher-order aberrations (μm)		0.16	0.08	0.17	0.10
Target IOL power (D)	142	17.33	5.11	17.31	5.05

UDVA, uncorrected distance visual acuity; UIVA, uncorrected intermediate visual acuity; UNVA, uncorrected near visual acuity.

Postoperative visual acuity outcomes indicated excellent performance at all follow-up time points. At the 3-months visit, the mean binocular UDVA was 0.017 ± 0.086 logMAR. The mean binocular UIVA at 60 cm was 0.061 ± 0.110 logMAR, and the mean binocular UNVA at 40 cm was 0.061 ± 0.103 logMAR. Repeated-measures analysis revealed that binocular UDVA and UIVA remained stable from postoperative day 1 through the 3-months follow-up (*P* > 0.05). In contrast, binocular UNVA improved significantly from postoperative day 1 (0.106 ± 0.148 logMAR) to the 1-week visit (0.053 ± 0.082 logMAR, *P* < 0.001) and remained stable thereafter (Table 3).

**TABLE 3:** Analysis of mean and standard deviation for binocular UDVA at 5 m, UIVA at 60 cm, and UNVA at 40 cm at various postoperative timepoints following PanOptix IOL implantation.

Parameter (LogMAR)	Follow-up time	Eyes (*n*)	Mean	SD	Skewness	Kurtosis
UDVA 5 m	1 d	134	0.019	0.081	0.053	0.115
	1 w		0.011	0.089	0.366	0.766
	1 m		0.027	0.117	4.424	34.475
	3 m		0.017	0.086	0.843	3.277
UIVA 60 cm	1 d		0.089	0.122	3.571	22.757
	1 w		0.073	0.087	0.555	1.073
	1 m		0.077	0.116	2.107	10.436
	3 m		0.061	0.110	1.295	2.526
UNVA 40 cm	1 d		0.106	0.148	3.500	18.715
	1 w		0.053	0.082	0.748	0.967
	1 m		0.060	0.093	0.983	1.136
	3 m		0.061	0.103	1.635	4.174

UDVA, uncorrected distance visual acuity; UIVA, uncorrected intermediate visual acuity; UNVA, uncorrected near visual acuity; logMAR, logarithm of the minimum angle of resolution; SD, standard deviation.

Analysis of the primary endpoint showed that 91.24% of patients achieved simultaneous binocular UDVA, UIVA, and UNVA of 0.200 logMAR or better at 3 months postoperatively. When each distance was assessed individually at the same follow-up visit, 98.51% of patients attained a UDVA of 0.200 logMAR or better, 93.28% achieved a UIVA of 0.200 logMAR or better, and 95.52% achieved a UNVA of 0.200 logMAR or better. Best-corrected visual acuity outcomes were likewise favorable: 96.85% of patients reached a BCDVA of 0.200 logMAR or better, 93.70% achieved a DCIVA of 0.200 logMAR or better, and 90.55% attained a DCNVA of 0.200 logMAR or better at 3 months.

Refractive predictability was high in this cohort. At 3 months postoperatively, 50.43% of eyes were within ±0.25 D of the intended refractive target, and 84.62% were within ±0.50 D. All eyes (100%) were within ±1.00 D of emmetropia (Table 4). Subjective refraction remained stable throughout the postoperative period, with no statistically significant differences in sphere or cylinder between the 1-week, 1-month, and 3-months visits (all *P* > 0.05).

**TABLE 4:** Distribution of postoperative refractive predictability (manifest refraction) at 1 week, 1 month, and 3 months following PanOptix IOL implantation.

Follow-up	Refractive error within target (D)	Binocular [*n* (%)]	OD [*n* (%)]	OS [*n* (%)]
1 week	±0.25	59 (50.43%)	81 (69.23%)	78 (66.67%)
	±0.50	96 (82.05%)	109 (93.16%)	100 (85.47%)
	±0.75	110 (94.02%)	114 (97.44%)	111 (94.87%)
	±1.00	114 (97.44%)	115 (98.29%)	115 (98.29%)
	±1.25	115 (98.29%)	116 (99.15%)	115 (98.29%)
	±1.50	116 (99.15%)	116 (99.15%)	117 (100.00%)
	±1.75	116 (99.15%)	116 (99.15%)	NA
	±2.00	117 (100.00%)	117 (100.00%)	NA
1 month	±0.25	62 (52.99%)	79 (67.52%)	76 (64.96%)
	±0.50	102 (87.18%)	110 (94.02%)	106 (90.60%)
	±0.75	115 (98.29%)	116 (99.15%)	115 (98.29%)
	±1.00	115 (98.29%)	117 (100.00%)	115 (98.29%)
	±1.25	117 (100.00%)	NA	117 (100.00%)
3 months	±0.25	59 (50.43%)	77 (65.81%)	74 (63.25%)
	±0.50	99 (84.62%)	107 (91.45%)	106 (90.60%)
	±0.75	112 (95.73%)	115 (98.29%)	114 (97.44%)
	±1.00	117 (100.00%)	117 (100.00%)	117 (100.00%)

D, Diopter. NA indicates that no additional eyes entered this cumulative threshold because all available eyes had already been included at a lower threshold.

**FIGURE 2:**
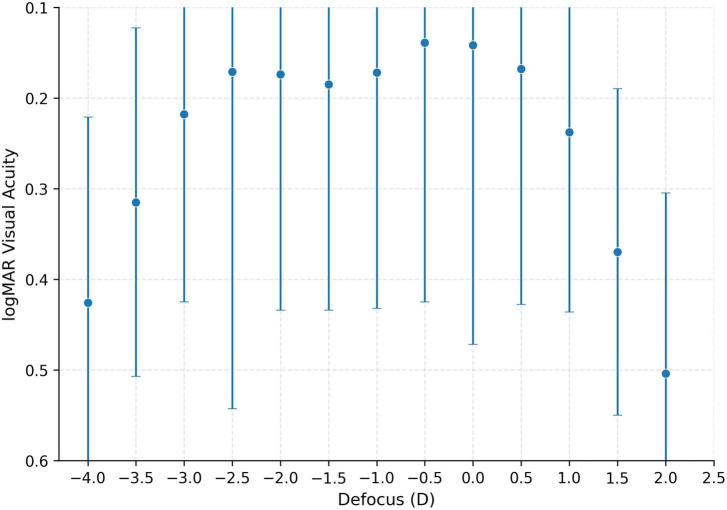
Binocular defocus curve at 3 months postoperatively. Data are presented as mean ± standard deviation of logMAR visual acuity at each defocus level from +2.00 D to −4.00 D.

The binocular defocus curve at 3 months postoperatively provided a comprehensive characterization of the functional range of vision. The curve demonstrated a broad plateau, with mean visual acuity better than 0.200 logMAR between +0.50 D and −2.50 D of defocus, effectively encompassing distances from optical infinity to approximately 40 cm (Table 5). Two principal peaks were observed: one at 0.00 D defocus, corresponding to distance vision, and a second at −2.50 D defocus, corresponding to near vision at 40 cm, with a highly functional intermediate range between −1.50 D and −2.00 D (Figure 2).

**TABLE 5:** Analysis of mean and standard deviation (SD) for monocular (OD and OS) and binocular defocus curves at 1 and 3 months postoperatively.

Follow-up	Eyes (*n*)	Refractive error within target (D)	OD		OS		OU	
			Mean	SD	Mean	SD	Mean	SD
1 month	124	+2.00	0.60	0.21	0.61	0.22	0.524	0.187
		+1.50	0.45	0.18	0.48	0.19	0.400	0.173
		+1.00	0.32	0.19	0.34	0.22	0.268	0.207
		+0.50	0.22	0.23	0.22	0.24	0.177	0.261
		+0.00	0.15	0.26	0.15	0.26	0.133	0.309
		−0.50	0.18	0.25	0.18	0.25	0.135	0.266
		−1.00	0.23	0.22	0.23	0.22	0.187	0.241
		−1.50	0.25	0.20	0.25	0.20	0.188	0.205
		−2.00	0.22	0.19	0.23	0.21	0.176	0.207
		−2.50	0.22	0.21	0.23	0.23	0.170	0.207
		−3.00	0.27	0.20	0.28	0.22	0.212	0.183
		−3.50	0.38	0.20	0.37	0.22	0.309	0.181
		−4.00	0.50	0.22	0.51	0.23	0.443	0.197
3 months		+2.00	0.58	0.22	0.57	0.22	0.504	0.199
		+1.50	0.44	0.21	0.44	0.19	0.370	0.180
		+1.00	0.30	0.20	0.30	0.19	0.238	0.198
		+0.50	0.22	0.24	0.21	0.23	0.168	0.260
		+0.00	0.16	0.28	0.15	0.28	0.142	0.330
		−0.50	0.18	0.26	0.18	0.27	0.139	0.286
		−1.00	0.22	0.22	0.23	0.24	0.172	0.260
		−1.50	0.24	0.22	0.23	0.22	0.185	0.249
		−2.00	0.23	0.22	0.22	0.23	0.174	0.260
		−2.50	0.23	0.22	0.23	0.23	0.171	0.372
		−3.00	0.27	0.20	0.29	0.22	0.218	0.207
		−3.50	0.39	0.20	0.40	0.23	0.315	0.192
		−4.00	0.50	0.22	0.53	0.24	0.426	0.205

D, Diopter.

Patient-reported outcomes assessed using the Catquest-9SF questionnaire indicated consistently high levels of satisfaction. At 3 months, 82.80% of patients reported no difficulty in performing daily activities due to their vision, and 87.50% reported being completely satisfied. Task-specific analysis showed excellent functional outcomes, with 95.30% of patients reporting no difficulty recognizing faces and 86.70% reporting no difficulty finding food labels.

Spectacle independence, evaluated with the IOLSAT questionnaire, was nearly complete. Overall, 96.90% of patients reported that they had not required spectacles for any distance over the preceding 7 days. In the small subset who used spectacles, they were predominantly required for near tasks under particular lighting conditions. Regarding overall satisfaction with the surgical procedure, 62.50% of patients were very satisfied and 28.10% were satisfied with their postoperative vision. Additionally, 94.50% indicated that they would choose the same IOL again, and 93.80% stated that they would recommend it to relatives or friends (Supplementary Tables 1–4).

Intraocular pressure demonstrated a statistically significant reduction over time, stabilizing at 3 months postoperatively at 13.80 ± 2.70 mmHg in the right eye and 13.90 ± 2.80 mmHg in the left eye. No sight-threatening adverse events attributable to the optical characteristics of the IOL were observed, and no lenses required explantation due to photic phenomena.

## 4 Discussion

This prospective multicenter registry study evaluated early visual performance and patient-reported outcomes after bilateral PanOptix implantation in Chinese cataract patients treated in routine ophthalmic practice. More than 91% of patients achieved binocular uncorrected visual acuity of 0.200 logMAR or better at distance, intermediate, and near simultaneously at 3 months, and the defocus curve showed a functional range of vision from distance to near. Our findings indicate that bilateral implantation of this non-apodized diffractive IOL yields excellent and stable visual acuity across the full spectrum of functional viewing distances. With more than 91% of patients achieving binocular logMAR visual acuity of 0.20 or better at distance, intermediate, and near simultaneously, the present study supports the robustness of the trifocal optical design in a heterogeneous clinical population.

The present study adds to the existing PanOptix literature in several respects. First, it provides prospective multicenter data from a Chinese cataract population, a group for which published real-world evidence remains relatively limited compared with Western cohorts. Second, the study used a composite primary endpoint requiring simultaneous binocular uncorrected visual acuity of 0.200 logMAR or better at distance, intermediate, and near, which is closer to patients’ daily functional visual demands than a single-distance endpoint. Third, the assessment combined clinical visual acuity outcomes, binocular defocus testing, refractive predictability, and patient-reported measures, allowing a broader evaluation of postoperative performance in routine practice.

When interpreted alongside studies of other presbyopia-correcting IOLs (3, 16, 17), the present findings appear consistent with the expected performance profile of a diffractive trifocal design. Previous comparative studies have shown that trifocal IOLs generally provide stronger near and intermediate visual performance than monofocal lenses, while EDOF lenses may offer a different balance between range of vision and photic symptoms. In a multicenter comparison of two trifocal presbyopia-correcting IOLs, both designs achieved good binocular visual acuity across functional distances, but differences in optical design and add power may influence the shape of the defocus curve and patient-reported visual experience (17). In our cohort, the broad binocular defocus plateau from +0.50 D to −2.50 D and the high rate of spectacle independence are in line with these published data, although direct comparison should be made cautiously because of differences in sample size, follow-up duration, measurement protocols, and patient selection.

The mean postoperative UDVA, UIVA, and UNVA of approximately 0.020, 0.060, and 0.060 logMAR, respectively, are comparable to or better than those reported in tightly controlled clinical trials performed in Western populations (18). These outcomes underscore the effectiveness of the ENLIGHTEN optical technology, which is designed to optimize light distribution while reducing dependence on pupil size (19). The high level of visual performance observed soon after surgery and sustained throughout the 3-months follow-up period suggests that this IOL delivers reliable and predictable refractive and visual outcomes, a feature that is critical for both clinician and patient confidence in presbyopia-correcting IOLs (20, 21).

A notable observation in this study is the significant improvement in UNVA between postoperative day 1 and the 1-week follow-up visit. This pattern implies that, whereas distance and intermediate visual acuity are restored almost immediately after surgery, near visual function may require a short period of neuroadaptation or ocular surface stabilization to reach optimal levels (22). Clinicians should consider this temporal profile when providing preoperative counseling and managing early postoperative expectations to avoid unnecessary concern regarding initial near visual performance. The consistent intermediate visual acuity at 60 cm observed throughout the follow-up period is particularly pertinent to the contemporary lifestyle of Chinese patients, who frequently engage in digital device use and other near-work activities at intermediate working distances (23).

The refractive predictability observed in this registry was high, with nearly 85% of eyes achieving a postoperative spherical equivalent within ±0.50 D of emmetropia. High refractive accuracy is a well-established prerequisite for the clinical success of multifocal and trifocal IOLs, as even low levels of residual spherical or cylindrical refractive error can significantly compromise image quality and increase the incidence of photic phenomena (20, 21). The multicenter nature of the present investigation, in which a range of biometry devices and surgical techniques were employed, further supports the robustness and generalizability of the refractive outcomes associated with this IOL when standard cataract surgery protocols and modern IOL power calculation methods are adhered to (24).

The binocular defocus curve offers a comprehensive representation of functional visual performance over a continuum of viewing distances. The smooth defocus profile and the extended plateau with visual acuity better than 0.200 logMAR between +0.50 D and −2.50 D indicate effective utilization of the IOL’s multiple focal points and support the optical optimization of the PanOptix design (25). The intermediate focal point at approximately 60 cm (corresponding to −1.66 D of defocus) effectively bridges the gap between distance and near foci, an interval that has traditionally represented a limitation of bifocal IOL designs (16). This optical configuration appears particularly well aligned with contemporary visual demands, including intermediate tasks such as computer use and smartphone interaction, which constitute primary functional priorities for many patients undergoing cataract surgery (26).

Patient-reported outcomes in this study were notably favorable, with nearly 97% of patients achieving complete spectacle independence. This degree of freedom from corrective lenses is a key determinant of patient satisfaction and is likely a major contributor to the high recommendation rate observed in this cohort (8). Although diffractive IOLs are known to be associated with photic phenomena such as halos and glare, our findings based on the Catquest-9SF and IOLSAT questionnaires indicate that these symptoms are generally well tolerated by most patients and do not substantially affect their daily functioning or overall satisfaction. The observation that more than 94% of patients would choose the same IOL again provides strong evidence of the perceived value of the full range of vision afforded by this lens design (17). The safety profile of the PanOptix IOL in this real-world setting was also favorable. The significant postoperative reduction in intraocular pressure, a commonly reported effect following cataract surgery, was sustained over the 3-months follow-up period (27). The absence of IOL explantations and of serious adverse events attributable to the optical properties of the lens further supports its safety as an option for presbyopia correction.

Despite these findings, this study has several limitations. First, the follow-up duration was limited to 3 months. This interval is sufficient to describe early postoperative visual recovery, refractive predictability, and initial patient-reported satisfaction, but it does not allow assessment of longer-term neuroadaptation, posterior capsule opacification, late-onset dysphotopsia, or the durability of spectacle independence. Second, the non-randomized registry design may have introduced selection bias, although it also reflects the performance of the IOL in routine clinical practice. Third, the study did not include a concurrent comparator group, and further comparative studies involving other trifocal and extended depth-of-focus IOLs in Chinese populations are needed. Fourth, 38 of the 175 enrolled patients did not complete the scheduled 3-months follow-up or had incomplete case-report documentation. Although available baseline comparisons did not indicate a clear major imbalance, residual attrition bias cannot be fully excluded, particularly because some baseline records were incomplete. Fifth, the inclusion of both age-related and complicated cataracts may have introduced clinical heterogeneity. All included patients met the same eligibility criteria for trifocal IOL implantation, and patients with ocular comorbidities likely to compromise postoperative outcomes were excluded; nevertheless, future studies with larger samples should perform stratified analyses by cataract subtype.

## 5 Conclusion

In conclusion, bilateral implantation of the AcrySof IQ PanOptix diffractive trifocal IOL was associated with favorable early full-range visual performance, high refractive predictability, and a high rate of spectacle independence among Chinese patients who completed 3 months postoperative follow-up. These outcomes, derived from a heterogeneous real-world clinical environment, offer evidence that this IOL is a highly effective option for patients seeking functional vision at all distances and minimization or elimination of dependence on spectacles following cataract surgery.

## Data Availability

The original contributions presented in this study are included in this article/Supplementary material, further inquiries can be directed to the corresponding author.
