# Supplementary material for: Visual performance and patient-reported outcomes of a non-apodized diffractive trifocal intraocular lens in Chinese cataract patients: a prospective multicenter real-world study
Source: Front Med (Lausanne). 2026 Jul 8;13:1853791. doi: 10.3389/fmed.2026.1853791 (PMC13388816; doi:10.3389/fmed.2026.1853791)
Supplement: Supplementary file 1 [file Data_Sheet_1.docx]

Supplementary Table 1 Analysis of the IOLSAT Spectacle Independence Questionnaire: Frequency of Wearing Glasses under Various Conditions

|  | Total participants | 5- Cannot decide（%） | 4- No difficulty（%） | 3- A little difficulty（%） | 2- Some difficulty（%） | 1- Very great difficulty（%） |
| --- | --- | --- | --- | --- | --- | --- |
| Does your sight at present cause you any difficulty in your everyday life? | 128 | 0  (0.00%) | 106  (82.80%) | 21  (16.40%) | 0  (0.00%) | 1  (0.80%) |
| Are you satisfied with your sight at present? |  | 0  (0.00%) | 112  (87.50%) | 15  (11.70%) | 0  (0.00%) | 1  (0.80%) |
| Does your sight at present cause you any difficulty reading text in daily newspapers? |  | 0  (0.00%) | 107  (83.60%) | 18  (14.10%) | 2  (1.60%) | 1  (0.80%) |
| Does your sight at present cause you any difficulty recognizing the faces of people you meet? |  | 0  (0.00%) | 121  (95.30%) | 5  (3.90%) | 1  (0.80%) | 0  (0.00%) |
| Does your sight at present cause you any difficulty seeing the prices or labels of goods when shopping? |  | 0  (0.00%) | 111  (86.70%) | 15  (11.70%) | 1  (0.80%) | 1  (0.80%) |
| Does your sight at present cause you any difficulty seeing to walk on uneven ground? |  | 0  (0.00%) | 123  (96.10%) | 3  (2.30%) | 2  (1.60%) | 0  (0.00%) |
| Does your sight at present cause you any difficulty seeing to do needlework, woodwork, or other fine activities? |  | 1  (0.80%) | 88  (68.80%) | 35  (27.30%) | 2  (1.60%) | 2  (1.60%) |
| Does your sight at present cause you any difficulty seeing to watch television or read subtitles? |  | 0  (0.00%) | 122  (95.30%) | 4  (3.10%) | 2  (1.60%) | 0  (0.00%) |
| Does your sight at present cause you any difficulty seeing to carry out an activity or hobby that you are interested in? |  | 0  (0.00%) | 124  (96.90%) | 3  (2.30%) | 1  (0.80%) | 0  (0.00%) |
