# Supplementary material for: Visual performance and patient-reported outcomes of a non-apodized diffractive trifocal intraocular lens in Chinese cataract patients: a prospective multicenter real-world study
Source: Front Med (Lausanne). 2026 Jul 8;13:1853791. doi: 10.3389/fmed.2026.1853791 (PMC13388816; doi:10.3389/fmed.2026.1853791)
Supplement: Supplementary file 2 [file Data_Sheet_2.docx]

Supplementary Table 2 Analysis of uncorrected visual acuity in different lighting conditions and distances based on the IOLSAT questionnaire

|  | Total Number (n) | 4-Very good（%） | 3-Good（%） | 2-Fair（%） | 1-Poor（%） | 0-Very poor（%） |
| --- | --- | --- | --- | --- | --- | --- |
| Please rate your vision when looking at objects at near distance (e.g., reading a book) without glasses in bright light during the past 7 days | 127 | 79  (62.20%) | 35  (27.60%) | 11  (8.70%) | 1  (0.80%) | 1  (0.80%) |
| Please rate your vision when looking at objects at near distance (e.g., reading a book) without glasses in dim light during the past 7 days |  | 47  (37.00%) | 41  (32.30%) | 35  (27.60%) | 4  (3.10%) | 0  (0.00%) |
| Please rate your vision when looking at objects at intermediate distance (e.g., using an ATM or viewing a car dashboard) without glasses in bright light during the past 7 days |  | 86  (67.70%) | 35  (27.60%) | 5  (3.90%) | 1  (0.80%) | 0  (0.00%) |
| Please rate your vision when looking at objects at intermediate distance (e.g., using an ATM or viewing a car dashboard) without glasses in dim light during the past 7 days |  | 63  (49.60%) | 41  (32.30%) | 23  (18.10%) | 0  (0.00%) | 0  (0.00%) |
| Please rate your vision when looking at objects at far distance (e.g., looking at street signs) without glasses in bright light during the past 7 days |  | 96  (75.60%) | 29  (22.80%) | 2  (1.60%) | 0  (0.00%) | 0  (0.00%) |
| Please rate your vision when looking at objects at far distance (e.g., looking at street signs) without glasses in dim light during the past 7 days |  | 75  (59.10%) | 38  (29.90%) | 14  (11.00%) | 0  (0.00%) | 0  (0.00%) |
