# Supplementary material for: Visual performance and patient-reported outcomes of a non-apodized diffractive trifocal intraocular lens in Chinese cataract patients: a prospective multicenter real-world study
Source: Front Med (Lausanne). 2026 Jul 8;13:1853791. doi: 10.3389/fmed.2026.1853791 (PMC13388816; doi:10.3389/fmed.2026.1853791)
Supplement: Supplementary file 3 [file Data_Sheet_3.docx]

Supplementary Table 3 Analysis of preoperative expectations for spectacles independence following cataract surgery based on the IOLSAT questionnaire

| Survey Item | Number of Participants | 4 - All of the time (%) | 3 - Most of the time (%) | 2 - Sometimes (%) | 1 - Rarely (%) | 0 - Never (%) |
| --- | --- | --- | --- | --- | --- | --- |
| How often did you expect to need to wear glasses to see objects at "near distance" (e.g., reading a book) after cataract surgery? | 128 | 0  (0.0%) | 3  (2.30%) | 8  (6.30%) | 14  (10.90%) | 103  (80.50%) |
| How often did you expect to need to wear glasses to see objects at "arm's length distance" (e.g., using an ATM or reading a car dashboard) after cataract surgery? | 128 | 0 (0.00%) | 0 (0.00%) | 11 (8.60%) | 11 (8.60%) | 106 (82.80%) |
| How often did you expect to need to wear glasses to see objects at "far distance" (e.g., reading road signs) after cataract surgery? | 128 | 0  (0.00%) | 0  (0.00%) | 6  (4.70%) | 7  (5.50%) | 115  (89.80%) |
