# Supplementary material for: Visual performance and patient-reported outcomes of a non-apodized diffractive trifocal intraocular lens in Chinese cataract patients: a prospective multicenter real-world study
Source: Front Med (Lausanne). 2026 Jul 8;13:1853791. doi: 10.3389/fmed.2026.1853791 (PMC13388816; doi:10.3389/fmed.2026.1853791)
Supplement: Supplementary file 4 [file Data_Sheet_4.docx]

Supplementary Table 4 Analysis of patient satisfaction with visual outcomes since cataract surgery based on the IOLSAT questionnaire

|  | Total Number (n) | 4-Very satisfied（%） | 3-Satisfied（%） | 2-Neither satisfied nor dissatisfied（%） | | 1-Dissatisfied（%） | 0-Very dissatisfied（%） |
| --- | --- | --- | --- | --- | --- | --- | --- |
| Overall, how satisfied have you been with your vision during the past 7 days? | 128 | 80  (62.50%) | 36  (28.10%) | 6  (4.70%) | | 1  (0.80%) | 5  (3.90%) |
|  | | Yes（%） | | | No（%） | | |
| Given your current vision, if you had to have the surgery again, would you choose to have the same type of intraocular lens implanted? | 128 | 121(94.50%) | | 7(5.50%) | | | |
| Given your current vision, would you recommend the intraocular lens you received to a family member or friend? |  | 120(93.80%) | | 8(6.30%) | | | |
